# Differences in Establishment of Persistence of Vaccine and Wild Type Rubella Viruses in Fetal Endothelial Cells

**DOI:** 10.1371/journal.pone.0133267

**Published:** 2015-07-15

**Authors:** Ludmila Perelygina, Adebola Adebayo, Maureen Metcalfe, Joseph Icenogle

**Affiliations:** Centers for Disease Control and Prevention, Atlanta, Georgia, United States of America; University of Georgia, UNITED STATES

## Abstract

Both wild type (WT) and vaccine rubella virus (RV) can pass through the placenta to infect a human fetus, but only wtRV routinely causes pathology. To investigate possible reasons for this, we compared establishment of persistence of wtRV and RA27/3 vaccine strains in fetal endothelial cells. We showed that yields of RA27/3 and wtRV were similar after the first round of replication, but then only vaccine-infected cultures went through a crisis characterized by partial cell loss and gradual decline of virus titer followed by recovery and establishment of persistent cultures with low levels of RA27/3 secretion. We compared various steps of virus replication, but we were unable to identify changes, which might explain the 2-log difference in RA27/3 and wtRV yields in persistently infected cultures. Whole genome sequencing did not reveal selection of virus variants in either the wtRV or RA27/3 cultures. Quantitative single-cell analysis of RV replication by in situ hybridization detected, on average, 1–4 copies of negative-strand RNA and ~50 copies of positive-strand genomic RNA in cells infected with both vaccine and WT viruses. The distinct characteristics of RA27/3 replication were the presence of large amounts of negative-strand RV RNA and RV dsRNA at the beginning of the crisis and the accumulation of high amounts of genomic RNA in a subpopulation of infected cells during crisis and persistence. These results suggest that RA27/3 can persist in fetal endothelial cells, but the characteristics of persistence and mechanisms for the establishment and maintenance of persistence are different from wtRV.

## Introduction

Rubella virus (genus Rubivirus, family Togaviridae) is a single-stranded RNA virus of positive genome polarity. WT rubella virus (RV) infection of susceptible women during the first trimester of pregnancy often results in long-term virus persistence in the fetus causing multiple organ abnormalities [1]. Yearly, there are an estimated 110,000 cases of congenital rubella syndrome (CRS) worldwide. Vaccination with live attenuated rubella vaccine is the most effective means to prevent CRS.

The rubella virus strain Wistar RA27/3 is currently one of the most used rubella vaccine viruses globally and one of the most successful vaccines ever developed [2, 3]. In addition to being ~ 97% effective in preventing clinical disease, vaccination with only a single dose induces both humoral and cellular immunity in > 95% of susceptible persons [4]. Most vaccinated persons had detectable rubella antibodies up to 20 years after one dose of rubella-containing vaccine [5]. Rubella outbreaks in populations vaccinated with the RA 27/3 are extremely rare. RA27/3 is so effective, that after 2001–2008 mass immunization campaigns, the Pan American Health Organization concluded based on surveillance data that the WHO Region of the Americas had eliminated rubella and CRS [6, 7]. Furthermore, the RA27/3 vaccine was shown to be safe and does not cause CRS [8]. Approximately 3,000 pregnant susceptible women were unknowingly immunized during early stages of pregnancy (≤4 gestational weeks (GW)) in mass campaigns in the Americas, but none of the infants had CRS as a result of vaccination [9].

There are two lines of evidence that suggest that RA27/3 can infect and persist in the fetus, detection of IgM antibody and RA27/3 virus in newborns. Follow-up studies after immunization campaigns discovered that 3.5% infants (70 out of 3000) born from inadvertently immunized pregnant women had detectable RV IgM antibody at birth [9]. Given that maternal IgM does not cross the placental barrier but can be produced by the fetus in response to intrauterine infections starting at 20–22 GW [10], the presence of rubella IgM in the newborn sera suggests that RA27/3 can cross the placenta with resultant congenital rubella infection (CRI), which can persist from ≤4 GW to at least 20 GW. To the best of our knowledge, the direct evidence of RA27/3 persistence in the fetus is limited to three reports, one of them documenting detection of RA27/3 virus in a product of conception by virus isolation [11] and the other two documenting detection of RA27/3 RNA in newborn specimens by RT-PCR and sequencing [12, 13]. On the other hand, many studies failed to detect RV27/3 genomes in oropharyngeal specimens from IgM-positive infants born from vaccinated mothers [9, 14, 15] and no infectious virus was recovered from fetal tissues obtained after maternal immunization [16]. It is presently unclear whether the low number of reports of RA27/3 persistence in newborns is attributable to the sensitivity of currently used methods or the inability of vaccine virus to persist to term in fetal tissues.

Cardiovascular defects are the leading cause of mortality among CRS patients [1, 17]. We have developed an *in vitro* model using primary fetal endothelial cells derived from human umbilical vein (HUVEC) to study vascular abnormalities in CRS [18]. We have demonstrated that the infection of HUVEC with clinical wtRV strains is not cytocidal and does not affect cell macromolecular synthesis or the cell cycle; wtRV was shown to persist at the single cell level.

The objective of this study was to determine whether RA27/3 vaccine rubella virus can infect and persist in HUVEC and whether the characteristics of persistence are different between wtRV and this vaccine virus.

## Material and Methods

### Cells and viruses

Human fetal vascular endothelial cells (HUVEC; cat# CC-2519, Lonza, Allendale, NJ) were maintained in Endothelial Growth Medium (EGM, Lonza, Allendale, NJ) replenished every 2–3 days and used between passages 3 and 5. HUVECs were derived from umbilical cord of the pregnancies, which were unlikely to have been effected by rubella, and the cultures are a pool of 20 donors. More than 99% of the cells in these cultures were positive by immunofluorescence for von Willebrand Factor (an endothelial cell marker). Thus, cells resulting from a host adaptive immune response to rubella virus were not relevant in these cultures. Vero cells (ATCC #CCL81) were maintained in DMEM/5% Fetal Bovine Serum (FBS). Vaccine strain Wistar RA27/3 (Serum Institute of India), and low passage clinical isolates RVi Dezhou.CHN 02 (RV-Dz, genotype 1E) and RVi Seattle.USA 16.00 (RV-WA, genotype 2B) were propagated in Vero cells and titered on Vero cells by immunocolorimetric plaque assay [19].

### Antibodies

The following mouse monoclonal antibodies were used: antibodies specific for rubella capsid protein (Abcam, Cat# ab34749), E1 protein (produced by CDC Core Facility), E2 (Meridian Life Science, Cat# C66497M), dsRNA-specific monoclonal antibody (J2, Scicons, Hungary), and HRP-conjugated β-actin-specific antibody (Sigma).

### Preparation of high titer virus stocks

High titer virus stocks of clinical isolates (passage 7 in Vero) and RA27/3 (passage 3 in Vero from the commercial monovalent vaccine stock) were prepared by concentrating infected cell media by tangential flow filtration using Biomax-300 cassette filter (Millipore, Bedford, MA) as previously described [18].

### Viral infections

HUVECs were seeded into wells of a 48-well plate at 1x10^5^ cells/well and infected with RV at MOI of 5 the following day. After 1-hour adsorption at 35°C, the monolayers were washed 3 times with Hank’s Balanced Salt Solution (HBSS) and overlaid with 0.5 ml of fresh media. The media and cultured cells were collected separately after 10 minutes incubation (0 hpi) and subsequent samples were collected at later times. For an infectivity assay, cell lysates were prepared by adding 0.5 ml media to the monolayers followed by 3 cycles of freeze-thaw. Virus amounts were determined by titering on Vero cells in duplicate. For viral RNA isolation, cell lysates was prepared by adding 350 μl of a kit RLT buffer (QIAgen) to the washed monolayers.

### Growth curve analysis of persistence

Cells were seeded into gelatin-coated T12 tissue culture flasks at 4x10^5^ cells/flask and mock infected or infected with RV at MOI of 5 or 0.05. After 1-hour adsorption at 35°C, the monolayers were treated with citrate buffer, pH 3.0, for 2 min to inactivate noninternalized virus. The monolayers were then washed 3 times with HBSS and overlaid with 2 ml media. Supernatants were collected after 10 minutes incubation (0 hpi) and subsequent samples were collected when media was changed every 2–3 days. Virus was titered on Vero cells in duplicate.

### Ultracentrifugation of viruses

Media collected from infected cells were precleared from cell debris by centrifugation at 350 x g for 10 min and then at 20,000 x g for 20 min. The precleared supernatant fluids (10 ml) were loaded on a cushion of 25% sucrose in HBSS (1.5 ml), followed by ultracentrifugation for 2 h at 35,000 rpm and 4°C using an SW41 rotor. The resulting pellets were resuspended in either RLT buffer (Qiagen) for RNA isolation or 1x SDS buffer (BioRad) for protein analysis.

### RNA Extraction and RT-qPCR

RNA was isolated from cell lysates using RNAeasy Mini kit (Qiagen) according to the manufacturer's instructions. Conditions for real-time qRT-PCR and the relative quantification of RV genomic RNA with the comparative threshold cycle (ΔΔ C_T_) method have been described earlier [18]. Absolute quantification of RV genomic RNA in the pelleted virions was performed by real-time qRT-PCR; assays were standardized using *in vitro* transcribed RV RNA standards [20].

### Western blot analysis

To analyze protein composition of the pelleted virions, the pellets were suspended in 50 μl of 1X non-reducing SDS sample buffer (BioRad, Richmond, CA), and then 10 μl of the RA27/3 preparation and 1 μl RV-Dz preparation were separated by 4–12% NuPage gels (Invitrogen, Carlsbad, CA). Different running buffers were used to separate proteins in conformations optimal for detection with each antibody, non-reducing (E1 MAb) or reducing (E2, C MAbs). To analyze viral proteins in infected cells, the cell monolayers were washed 3 times with ice-cold PBS and then proteins were extracted with RIPA buffer (Thermo Scientific, Rockford, IL) supplemented with Halt protease cocktail (Thermo Scientific, Rockford, IL). Protein concentration in the extracts was determined by BCA assay (Thermo Scientific, Rockford, IL). Equal amounts of total protein (10 μg/lane) for each sample were separated by 4–12% NuPage gels (Invitrogen, Carlsbad, CA) using MOPS running buffer and then transferred onto nitrocellulose membrane. SNAP i.d. Protein Detection System (Millipore) was used to process western blots as described earlier [18]. Blots were developed with the ECL-plus detection reagents (GE Healthcare, Piscataway, NJ) and exposed to X-ray film. The intensity of capsid band was quantified by densitometry with Carestream Molecular Imaging Software (Carestream Health Inc., New Haven, CT).

### Immunofluorescence analysis (IFA)

Cells were rinsed with PBS, fixed with -20°C methanol for 10 min and blocked for 1 h at room temperature in 3% BSA-PBST (PBS-0.1% Tween-20). The cells were stained with primary monoclonal antibody for 1 h at room temperature followed by incubation with anti-mouse IgG Alexa Fluor 488 (Invitrogen, Carlsbad, CA). Nuclei were counterstained with DAPI. Cells were mounted with fluorescence mounting medium (Dako, Carpinteria, CA). Images were acquired using a Zeiss fluorescent microscope. To estimate the percentage of stained cells, positively stained cells and total nuclei were counted in at least four microscopic fields (total ~200–300 cells).

### *In situ* hybridization

Cells were grown on poly-lysine coated chamber slides (BD Biosciences) additionally coated with gelatin. The QuantiGene ViewRNA assay kit (Affymetrix, Cat # QV0096) was used for detection of RNA hybridization signals. Cells were fixed with 4% paraformaldehyde for 30 min, permeabilized with detergent solution for 5 min and then treated with protease for 10 min at room temperature. Each hybridization step, which was carried out in a hybridization oven at 40°C, was followed by three washes with wash buffer. Initially cells were incubated in an assay kit hybridization solution A containing a probe set for either the negative or positive stranded RNA rubella genome (complementary to the 5’ genomic region spanning nucleotides 1 to 6000) labeled with Cy5 (Affymetrix, custom made) in combination with a probe set for FITC-labeled housekeeping gene, peptidylpropyl isomerase B (PPIB, Affymetrix, Cat # VA4-10510), or a negative control probe set against Influenza A virus NP-negative strand labeled with Cy5 (Affymetrix, Cat# VF1-10583) in combination with a PPIB probe set. The cells were then incubated with hybridization preamplifiers and finally with labeled probes (1:100 in an assay kit hybridization buffer C), washed and imaged using a Zeiss fluorescent microscope. AxioVision software was used to create an outline of a cell periphery based on PPIB localization.

### Sequencing

A detailed description of the full-genome sequencing strategy for rubella virus has been published earlier [21].

### Transmission electron microscopy

Cells were infected with RV at an MOI of 50. Cell culture pellets were collected at 24 hpi and fixed with 2.5% glutaraldehyde. Further sample processing and analysis by electron microscopy has been described in detail [18].

### Statistical analyses

The one-way analysis of variance (ANOVA) test with post hoc analysis using the Tukey test for multiple comparisons was used to compare differences between number of positive and negative strand RV RNAs in cells infected with wtRV and vaccine virus. A *P* value of <0.05 was considered significant. Statistical analyses were performed using the GraphPad Prizm 5 software (GraphPad Software, San Diego, CA).

## Results

**Fig 1:**
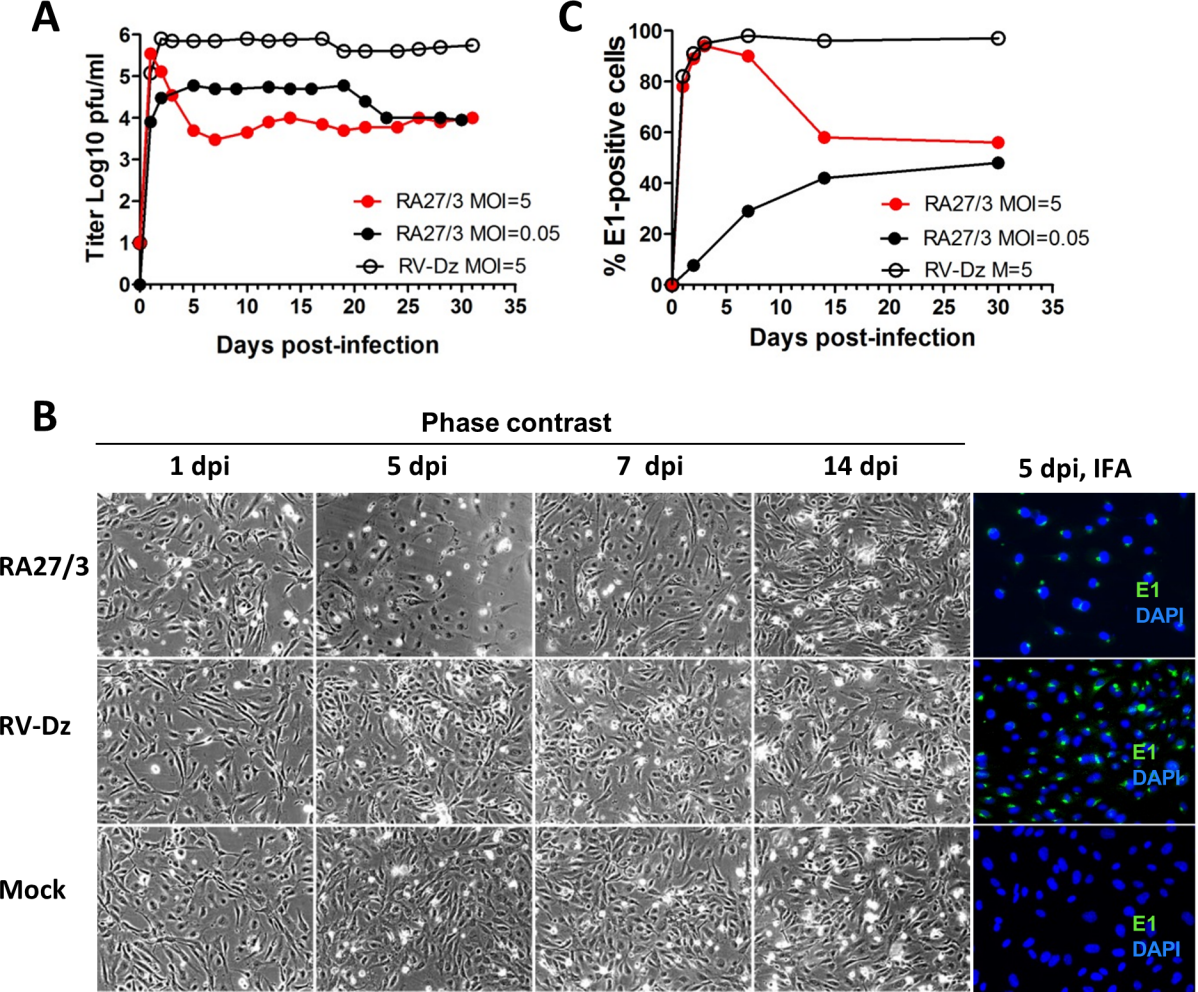
Comparative analysis of vaccine and wtRV persistence in HUVEC. (A) Growth curves of RA27/3 and RV-Dz at different MOIs. (B) Percentage of E1-positive cells in the infected monolayers at different times post-infection. Representative results of at least two independent experiments are shown. (C) Phase contrast images and results of immunofluorescence assay (E1 staining) of HUVEC infected with RA27/3, RV-Dz or mock-infected at different times post-infection. Nuclei were counterstained with DAPI.

The establishment of persistence in fetal endothelial cells of the RA27/3 vaccine and WT strains of RV was compared in parallel HUVEC cultures infected with RA27/3 or RV-Dz virus at MOI = 5 and maintained without cell passage for one month. The titer of extracellular virus was determined every 2–3 days, when media were changed, by titration on Vero cells. At 1 dpi, RA27/3 and RV-Dz viruses produced comparable titers, but after that their growth curves were dramatically different (Fig 1A). In agreement with the previously published results, the RV-Dz virus production reached a maximum at 2 dpi and remained at approximately the same level until culture senescence. In contrast, the vaccine virus-infected cultures went into a lytic crisis lasting from the day 2 to 5. During this period we observed a gradual 2-log reduction of the vaccine virus titer (from 5x10^5^ to 5x10^3^) concurrent with considerable cytopathic effect (CPE) resulting in 50–70% cell loss (Fig 1B). The vaccine virus-infected monolayers then restored and themselves became indistinguishable morphologically from the mock and RV-Dz infected monolayers at 7 dpi, but the amount of RA27/3 virus continued to persist at the same low level (Fig 1A and 1B). The results indicate that, unlike non-lytic WT rubella viruses, RA27/3 vaccine virus kills infected endothelial cells. Nonetheless, after culture crisis, RA27/3 persistent cultures with low virus yields were established. These data suggest that different mechanism(s) regulate the establishment and maintenance of persistence of RA27/3 vaccine and wtRV in HUVEC.

At least three possible underlying mechanisms could explain the establishment of RA27/3 persistent HUVEC cultures with the observed characteristics. First of all, the vaccine virus stock might contain a mixture of cytopathic and non-cytopathic variants of RA27/3 virus and a non-cytopathic variant was selected after the culture crisis. To test this hypothesis, we compared the complete genomic sequences of the RA27/3 stock virus and the virus isolated from the HUVEC persistent culture at 14 dpi. The sequences of these viruses were identical. Interestingly, heterogeneity detected in the two genome positions 1967 (A/G, 643K/643E in the p150 nonstructural protein) and 3571 (A/G, synonymous substitution) in the stock virus was maintained in the virus isolated after the crisis. Whether the heterogeneity was present in the commercial RA27/3 preparation could not be examined because the entire vial content was used for stock preparation. The published RA27/3 sequences have an A residue in a position 1967 and G residue in a position 3571, which are conserved in the rubella genome [21–23]. Since genomic sequences of RA27/3 did not change after the crisis, including at the two heterogeneous positions, we concluded that RA27/3 persistence in HUVEC was not established by a selection of a less cytopathic variant. Similarly, the consensus genomic sequence of RV-Dz remained stable in persistently infected HUVEC cultures [18].

A second possibility is that RA27/3 has a defect in cell entry and does not infect the entire monolayer at MOI = 5. Since RA27/3 is cytocidal in HUVEC, the infected cells would produce a large virus titer and die after an initial round of RV replication while the remaining uninfected cells proliferate and become the dominant cells in the culture. Since medium must be replaced every 2–3 day in HUVEC cultures, the restored uninfected monolayers would be exposed to low amounts of RA27/3 and, therefore, only a small percentage of infected cells might continue to persist and produce small quantities of the virus after the crisis. To assess this possibility, we estimated the number of infected cells by immunofluorescence staining of the infected monolayers for E1 protein at different times post-infection. Similar to RV-Dz infected HUVECs, almost all cells (>90%) were E1-positive in the RA27/3-infected monolayers from 2 to 5 dpi (Fig 1C). These data indicate that cell entry of RV-Dz and RA27/3 into HUVEC was equally efficient and demonstrate that the crisis and virus titer drop observed in the culture infected with the vaccine virus occurred without significant reduction of the proportion of infected cells. After the crisis, the number of E1-positive cells decreased to ~50% at 14 dpi in vaccine infected monolayers while remaining constant (>95%) in RV-Dz infected HUVEC (Fig 1C). Almost all cells in the both monolayers showed evidence of infection at 14 dpi by in situ hybridization (see below).

To investigate whether vaccine infected cultures undergo the same crisis after a low MOI infection, we infected HUVEC with RA27/3 at an MOI of 0.05 and performed an analysis as described above. Minimal cell rounding and detachment occurred at the similar rates in both mock-infected and vaccine-infected monolayers. As expected, a small number of cells were infected initially (7%) and by two weeks post-infection the percentage of E1-positive cells (~40%) became similar to that in HUVEC infected with RA27/3 at high MOI (Fig 1C). Virus production reached maximum at day 2 (5x10^4^ pfu/ml) and remained at this level for 15–20 days, but then the titer was reduced to the level seen in the culture infected with high MOI (Fig 1A). No crisis was detectable after low MOI inoculation with vaccine. Cells infected with low MOI and high MOI (after crisis only) can be passaged and the same low level of virus production (10^3^–10^4^) remained after three sequential passages during two weeks of cultivation (data not shown). Thus, the overall characteristics of persistent cultures (e.g. at 30 dpi) derived from low and high RA27/3 MOI infections were the same.

**Fig 2:**
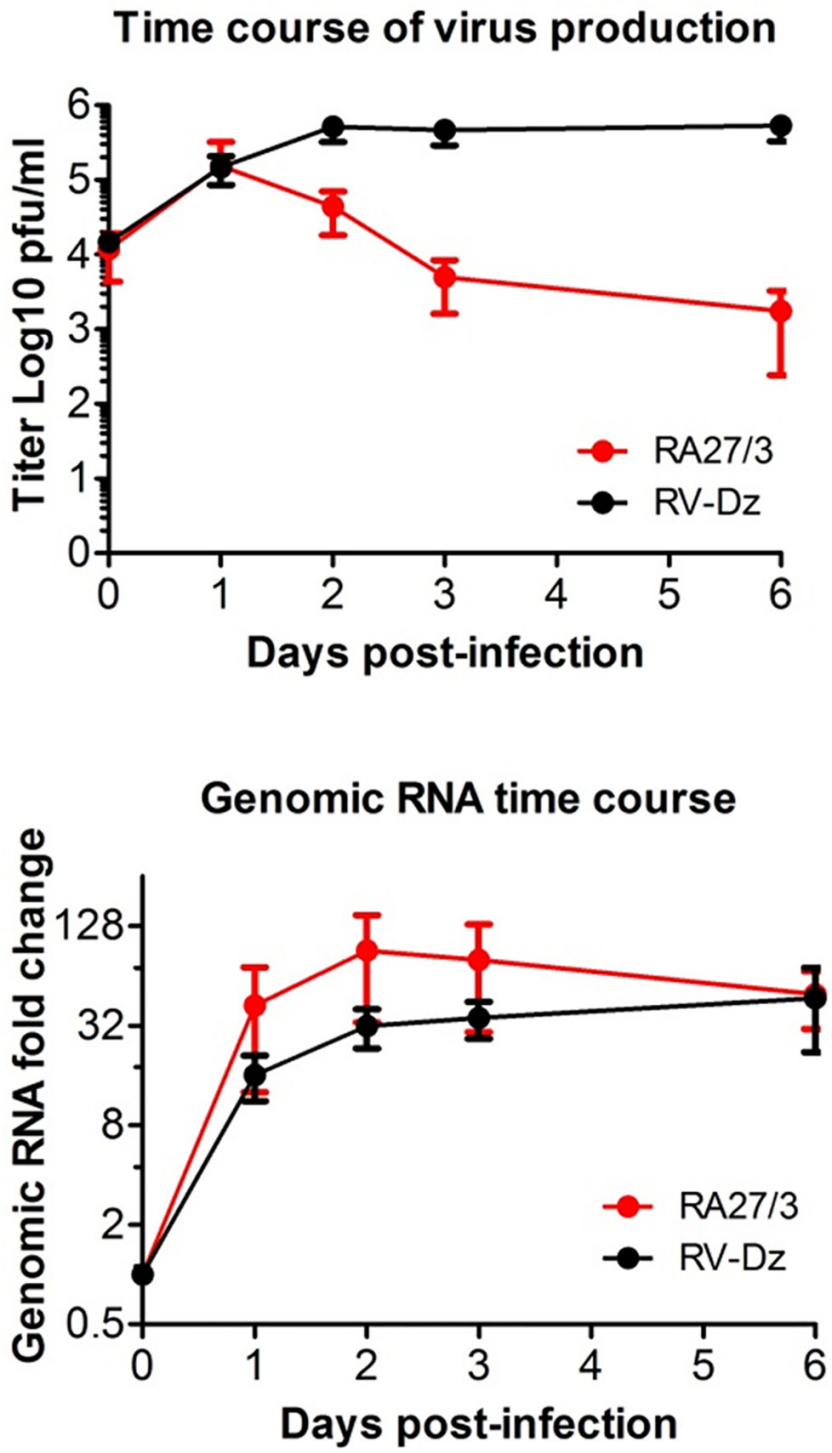
Kinetics of virus production and genomic RNA replication. HUVECs were infected with RA27/3 and RV-Dz at an MOI of 5. (A) Quantitation of infectious extracellular virions by titering the cell culture supernatants on Vero cells. (B) Quantitation of intracellular rubella genomic RNA by RT-qPCR with the ΔΔ C_T_ method using GAPDH mRNA for normalization. Data are presented relative to the genomic RNA amount at 4 hpi before the beginning of detectable replication. Data for (A) and (B) are presented as a mean +/- SD of three independent experiments.

A third possible mechanism of establishment of RA27/3 persistence is the reduction of RA27/3 yield to the level that could be tolerated by HUVEC. This could be mediated by cellular antiviral defenses, which target various steps of a virus replication cycle, such as viral RNA replication, viral protein production, particle assembly or egress. To determine if the production of genomic RNA was inhibited in the vaccine virus infected cells, we studied the kinetics of genomic RNA replication during the crisis by quantitation of RV genomic RNA in the infected cells with real-time qRT-PCR relative to 4-h time point [18]. The titers of infectious viruses in the culture media were also determined. In the RV-Dz infected HUVECs, the kinetics of virus production and genomic RNA replication were similar (Fig 2). In the vaccine virus infected cells, the infectious virus production was reduced by 2 logs relative to RV-Dz, but RNA replication was comparable to that of RV-Dz. These data demonstrate that the reduction of vaccine virus titer during the culture crisis was not a result of the inhibition of RA27/4 RNA replication in HUVEC.

To assess RV replication on a single cell level, we used RNA fluorescence *in situ* hybridization (RNA-FISH). This assay uses branched fluorescently labeled DNA probes and offers single molecule sensitivity [24]. We used a FITC-labeled PPIB probe set as a positive control and a Cy5-labeled Flu NP probe set as a negative control. We observed ~150–250 dots per cell for the PPIB probe set and less than 1 dot/cell for the Flu NP probe set, which is consistent with the reported levels of the PPIB gene expression and non-specific background (Affymetrix technical support, personal communication). These data verified high sensitivity and specificity of the assay in our hands.

**Fig 3:**
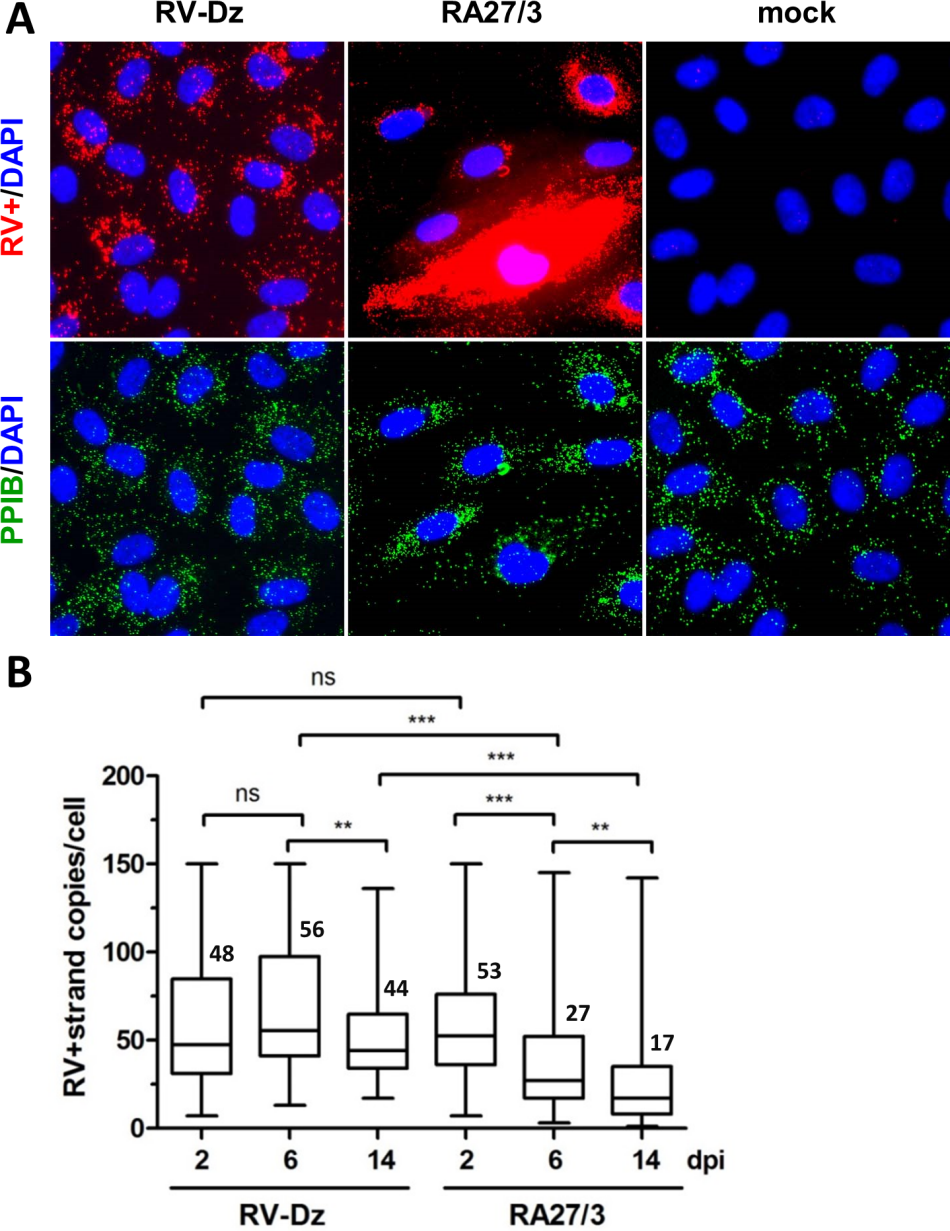
Quantitation of positive-strand RV RNA by *in situ* hybridization. (A) Representative images from at least 3 independent experiments showing the results of the RNA-FISH for positive-strand RV RNA in HUVECs mock infected or infected with RA27/3 or RV-Dz at 6 dpi. Outlines of cell boundaries were created in the AxioVision software (Zeiss, Germany) by using PPIB dots as reference points. Note the presence of cells with high hybridization signal in RA27/3 infected HUVECs. (B) Distribution of dots (plus-strand genomic RNA) per cell in infected HUVECs with countable signal (up to 150 dots/cell). The dots were counted in 7–10 randomly selected microscopic fields (total 120 cells). Combined data from two separate experiments are presented in a box-and-whisker plot such that the edges of the boxes represent the 25th and 75th percentiles, the horizontal line in the box is the median, and the whiskers show the range of values. Significance was determined using ANOVA, followed by post hoc analysis using the Tukey test for multiple comparisons (**, P < 0.01; ***, P < 0.001).

HUVECs were mock-infected or infected with RA27/3 or RV-Dz at an MOI of 5. We selected three time points for RNA-FISH analysis: 2 dpi (the beginning of the crisis), 6 dpi (the end of the crisis), and 14 dpi, when the persistent cultures were stable. RV RNA was quantified by counting dots in 120 randomly selected cells for each infection at the three time points after hybridization of monolayers with either the negative- strand or positive-strand specific RV probe sets labeled with Cy5. RV RNA was identified as bright discrete dots dispersed in cytoplasm with some accumulation in the perinuclear region. Representative RNA-FISH results using the RV positive-strand probe set are shown in Fig 3A. All cells in both WT and vaccine infected monolayers contained positive-strand RV RNA at 2, 6 and 14 dpi indicating that all cells were infected. While bulk genomic RNA production was the same in WT and vaccine virus infected HUVECs (Fig 2), the variation in the amount of the positive-strand RV RNA per cell was much higher in the cells infected with vaccine virus than with WT viruses (Fig 3A). Three cell populations were distinguishable in vaccine virus infected cells—low (less than 150 dots/cell), medium (150–250 dots/cell) and high RNA producers (more than 250 dots/cell). Table 1 shows the percentage of cells in each category. For both viruses, there was a similar tendency to reduce the number of medium RNA producers. The cells with high amounts of positive-strand RNAs were almost exclusively detected in the vaccine virus infected cells, not in the monolayers infected with RV-Dz or the other WT strain RV-WA (Fig 3A and data not shown). The proportion of these cells did not change during the crisis and reduced only 2-fold after the crisis. On the other hand, the majority of cells in both monolayers contained less than 150 copies of positive-strand RNA per cell (Table 1). The average number of positive-strand RNA per cell in this category did not change significantly in RV-Dz infected HUVEC during persistence and reduced only 2–3 fold in vaccine-infected HUVEC (Fig 3B). Thus, while quantitative analysis of genomic RNA on a single cell level provided evidence of significant differences in the number of cells with high levels of positive strand RNA, it did not provide us with an insight into causes of the 2-log titer reduction of vaccine virus.

**Table 1:** Proportion of cells (%) with different amounts of positive-strand genomic RNA in RA27/3 and RV-Dz infected HUVEC.

Time	RA27/3*			RV-Dz*		
	<150	150–250	>250	<150	150–250	>250
**2 dpi**	65.9	22.7	11.4	77.4	22.6	0
**6 dpi**	79.9	9.4	10.7	93.3	6.7	0
**14 dpi**	88.0	8.0	4.0	98.4	1.6	0

*At least 500 cells were analyzed for each infection at the each of three time points.

**Fig 4:**
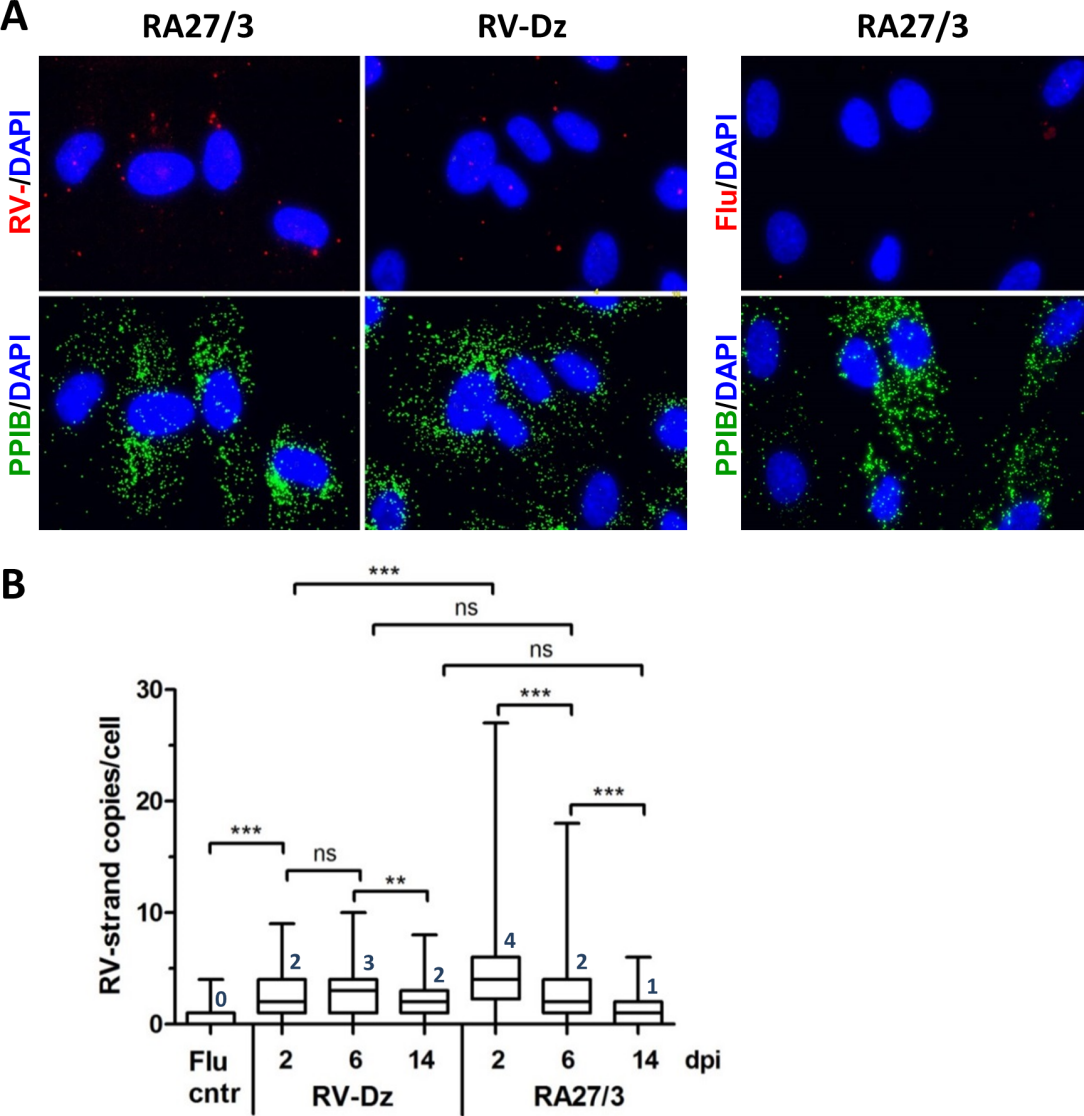
Quantitation of negative-strand RV RNA by *in situ* hybridization. (A) The representative images from at least 3 independent experiments showing the results of the RNA-FISH for negative-strand RV RNA and influenza NP-negative strand RNA (negative control) in HUVECs infected with RA27/3 and RV-Dz at 2 dpi. Outlines of cell boundaries were created in the AxioVision software by using PPIB dots as reference points. (B) Distribution of the dots (negative-strand genomic RNA) per cell in infected HUVEC. The dots were counted in 7–10 randomly selected microscopic fields (total 120 cells). The combined data from two separate experiments are presented in a box-and-whisker plot such that the edges of the boxes represent the 25th and 75th percentiles, the horizontal line in the box is the median, and the whiskers show the range of values. Significance was determined using ANOVA, followed by post hoc analysis using the Tukey test for multiple comparisons (**, P < 0.01; ***, P < 0.001).

The representative FISH results using the negative-strand RV probe set are shown in Fig 4A. Box-and-whisker plot (Fig 4B) shows the distribution of RV negative- strand copies per cell at the different times post-infection for both viruses. To our surprise, low amounts of negative-strand RNA template were produced (1–4 copies per cell on average) and they were essentially the same in both RV-Dz and vaccine virus infected cells at 6 and 14 dpi. The only significant difference between vaccine and WT infected cultures was the presence of cells with elevated amounts of negative-strand RNA in the RA27/3-infected monolayers at 2 dpi (P<0.001). The median amounts of negative-strand RV RNA per cell were reduced by 14 dpi in vaccine infected HUVEC, but unchanged in RV-Dz infected cells suggesting negative-strand RV RNA stability of RA/27 and RV-Dz might be different.

**Fig 5:**
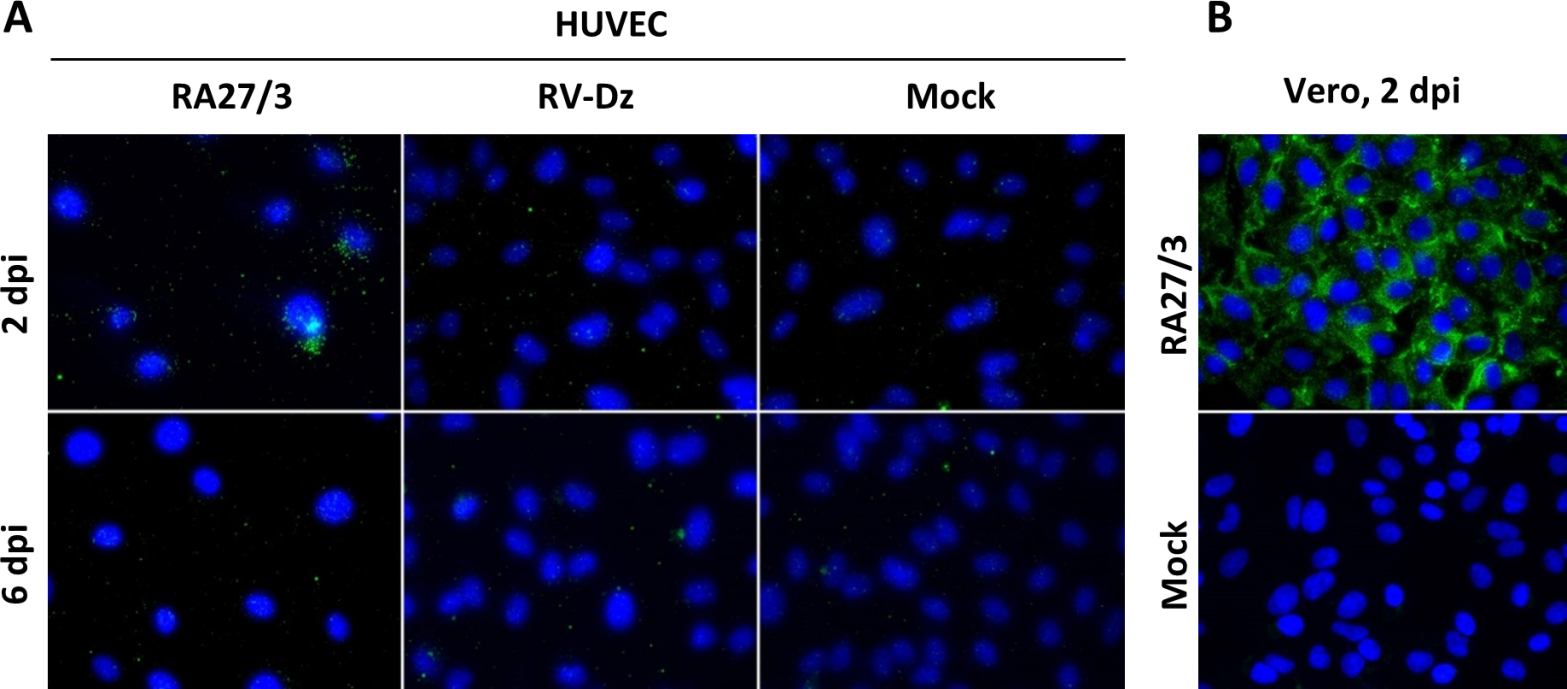
Detection of RV dsRNA in infected cells by immunofluorescence. RV infected and mock-infected HUVECs (A) or Vero cells (B) were fixed with methanol and examined for presence of dsRNA by indirect immunofluorescence using dsRNA-specific antibody. Nuclei were counterstained with DAPI.

Negative-strand templates exist in a cell mainly in a form of dsRNA, which serves as a marker to detect replication complexes of positive-strand viruses [25–27]. Staining of the infected HUVECs with a dsRNA-specific antibody demonstrated that virus-specific dsRNA accumulated in 53.8% of RA27/3 infected cells (86 out of 160) at 2 dpi but became undetectable at 6 dpi (Fig 5A). The staining was punctuate with less than 30 stained dots per cell and appeared to accumulate mainly in the perinuclear area. RV-Dz and mock infected monolayers, dsRNA was not detectable at either time point. Consistent with the published results [25], dsRNA was easily detectable in rubella virus-infected Vero cells, which served as a positive control for antibody (Fig 5B). These data suggest that the HUVECs with elevated amounts of negative-strand RV templates and dsRNA most probably represent the cells that were dying during the crisis in RA27/3 infected cultures (Fig 1). Cell death, however, could account only for ~0.5 log reduction in RA27/3 virus yield.

**Fig 6:**
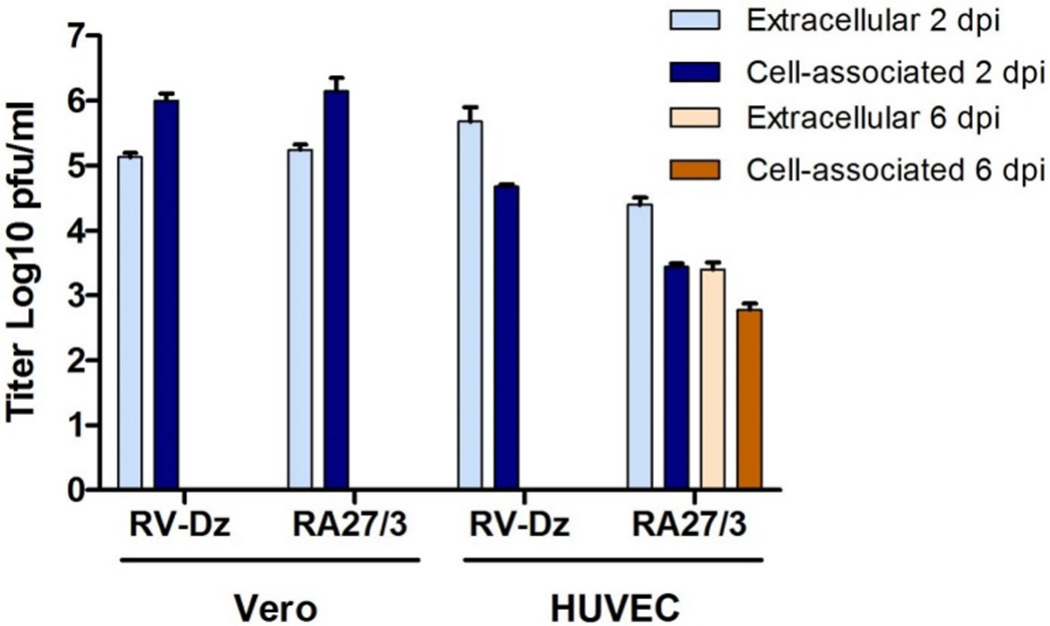
Secretion of infectious rubella virions into growth media. Vero and HUVEC were infected with RA27/3 and RV-Dz at MOI = 5. Media and cells were collected at 2 dpi and titered on Vero cells. Note that secretion of WT and vaccine viruses from HUVEC were equally effective.

Next, we investigated if post-RNA replication steps were different in the vaccine virus infected cells. We have shown previously that wtRV egress was efficient from HUVEC, but ~90% of infectious virus remained cell-associated in Vero cells [18]. It is possible that, similar to WT rubella viruses in Vero cells, the interaction of RA27/3 virions with the secretory machinery of endothelial cells was ineffective. To evaluate the efficiency of RA27/3 egress, we determined titers of the extracellular and cell-associated infectious virions at 2 and 6 dpi and compared to those of RV-Dz in the infected HUVEC and Vero cells at 2 dpi. The data presented in Fig 6 indicate that secretion of RV-Dz and RA27/3 was equally effective from HUVEC and similarly ineffective from Vero. These data indicate that inefficient egress from HUVEC is not the cause of reduction of RA27/3 virus production compared to wtRV during the crisis.

**Fig 7:**
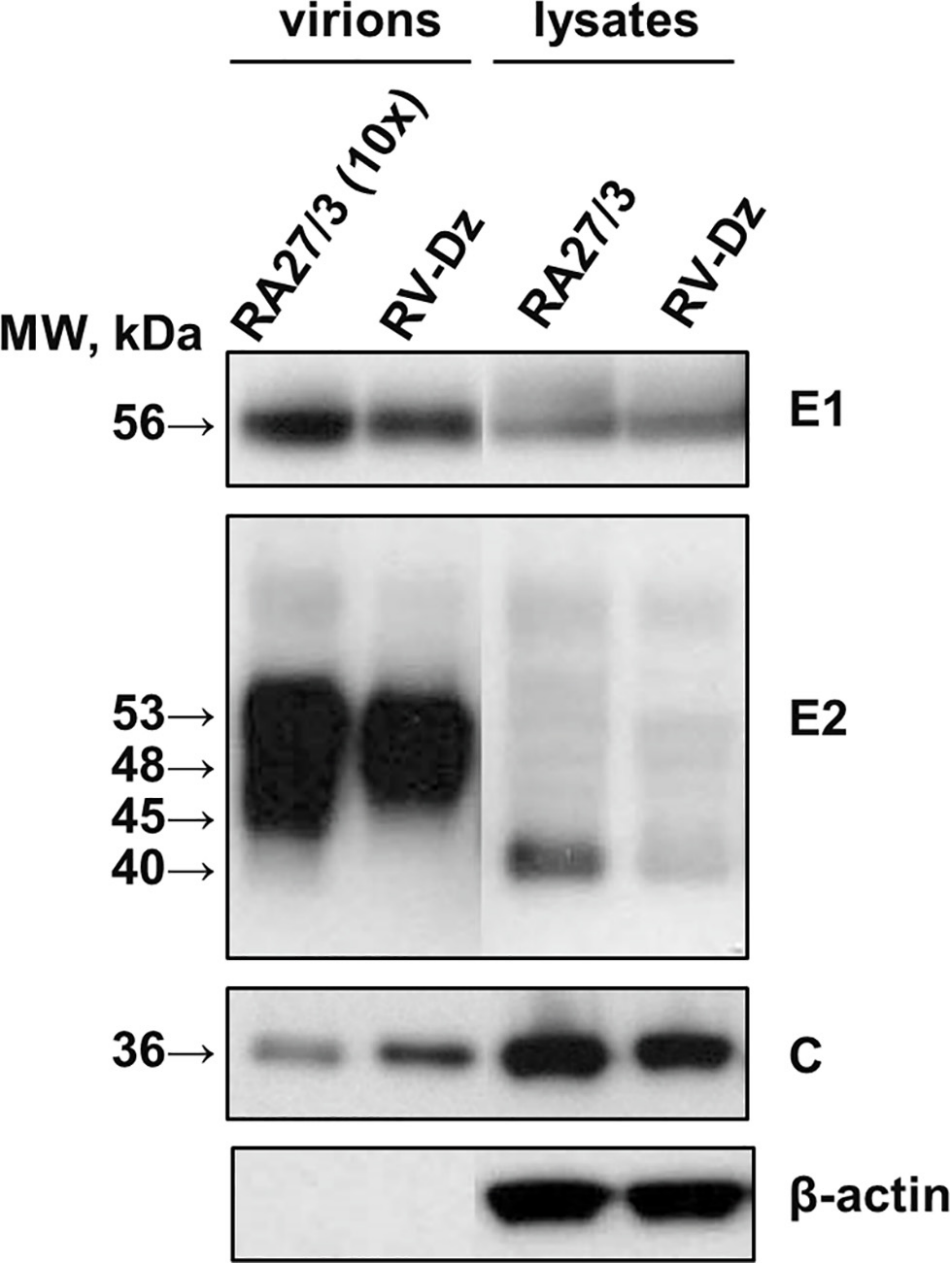
Western blot analysis of purified vaccine and wtRV virions and viral proteins in the infected HUVEC. Vero and HUVEC were infected with RA27/3 and RV-Dz at MOI = 5. Virions were pelleted from the culture media by ultracentrifugation and resuspended in an SDS-sample buffer. The cell monolayers were washed with PBS and proteins were then extracted with RIPA buffer. Proteins were separated by a 4–12% NuPage gel, either nonreducing (E1, C, β-actin) or reducing (E2), and then the blots were probed with rubella E1, E2 and C-specific MAb to identify RV structural proteins. The blots were also probed with β-actin MAb to demonstrate equal protein loading for the analysis of the cell lysates and show purity of the pelleted virions. Representative results of two independent experiments are shown.

Another possibility is that the same virion quantities were produced in vaccine and WT infected HUVEC cultures, but the majority of RA27/3 particles were noninfectious. Particle-to-pfu ratio for rubella virus has never been reported. The direct estimation of particle-to-pfu ratio of the virions in media by thin section transmission electron microscopy is problematic for RV because of low virus titer. We used a different approach and measured genomic RNA-to-pfu ratio for virions purified from culture media collected at 3 dpi from RV-Dz and RA27/3 infected HUVECs. Since the infectivity of fragile rubella virions was shown to be greatly affected by ultracentrifugation [28], we measured total infectivity of the media prior to pelleting virions by ultracentrifugation through a sucrose cushion (Table 2). Western blot analysis of the pellets revealed the lack of ß-actin bands in the virion preparations thus confirming efficient virion purification from cellular proteins (Fig 7). The total amount of genomic RNA in the pelleted virions was determined by qRT-PCR (Table 2). The data demonstrate that the genomic RNA-to-pfu ratio was the same for vaccine and WT viruses.

**Table 2:** Genomic RNA (gRNA)-to-pfu ratio for RA27/3 and RV-Dz virions produced in HUVEC.

Virus	gRNA total	Pfu total	gRNA/pfu
**RA27/3**	4.2E+07	1.2E+06	35.4
**RV-Dz**	7.9E+08	2.0E+07	39.7

To exclude the possibility that the vaccine virus preparation contained more defective virions lacking genomic RNA compared to the RV-Dz preparation, capsid amounts were estimated by Western blot. The ratio of RV-Dz to RA27/3 genomic RNA and ratio of RV-Dz to RA27/3 capsid protein amounts were comparable, 18.8 and 17.1, respectively. In addition, Western blot analysis showed that there were no substantial differences in the protein composition of WT and vaccine rubella virions and in production of the rubella structural proteins in the infected cells (Fig 7). Differences in the intensity and mobility of some E2 bands were most likely related to the variability of carbohydrate moiety of this highly glycosylated RV protein [29] emphasizing the importance of using the C protein for this comparison. These data indicate that if defective particles were produced, they were present in similar proportions in RA27/3 and RV-Dz infected HUVEC.

**Fig 8:**
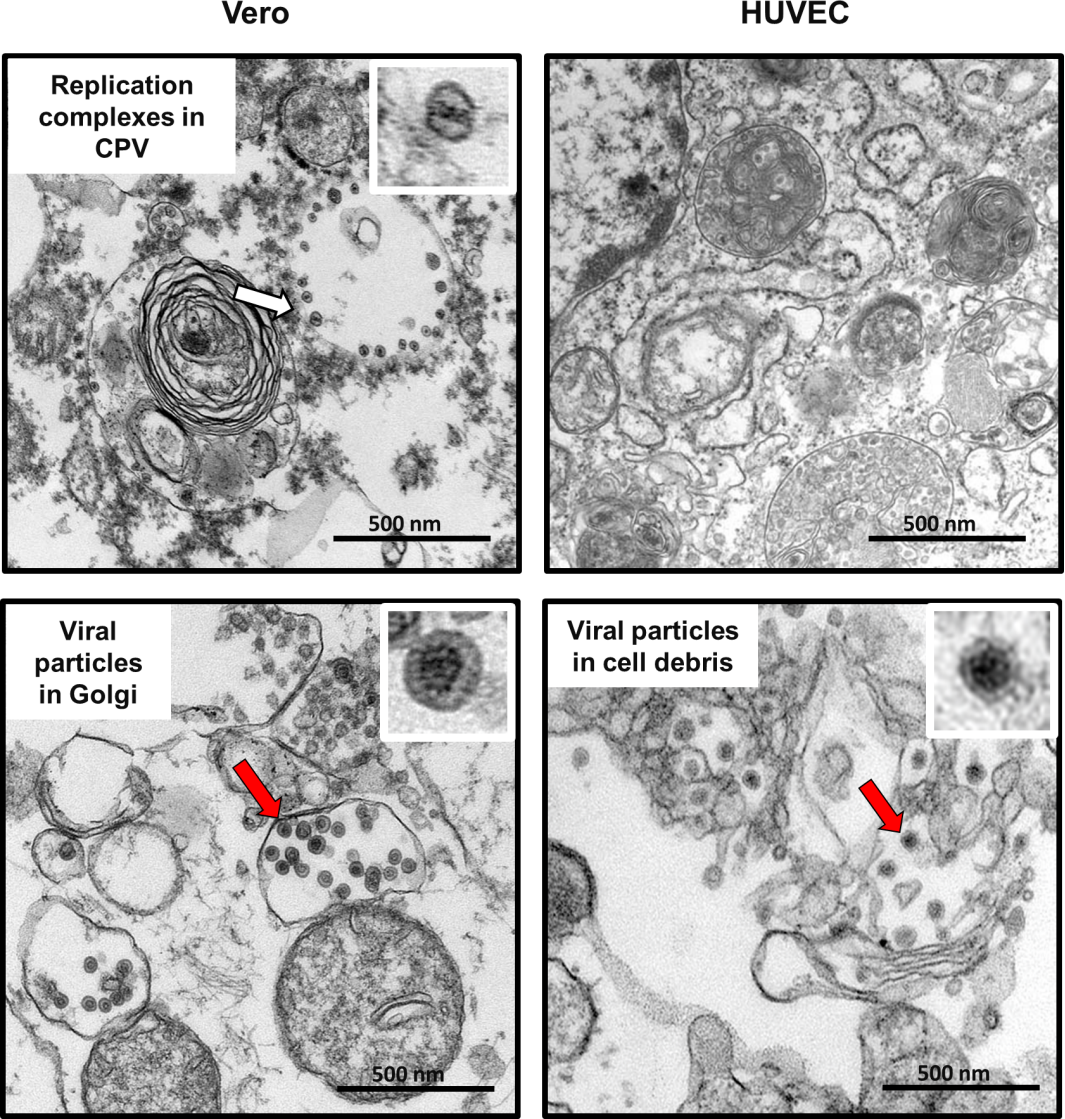
Rubella particles and replication complexes in RA27/3 infected HUVEC and Vero cells. Representative images of rubella virions (the red arrows) and replication complexes (the white arrow) in cytopathic vacuoles (CPV) observed by transmission electron microscopy in HUVEC and Vero cells infected with RV-Dz at MOI = 50 at 24 hpi. Inserts represent enlarged images of the replication complex and virions that are marked with the arrows. Scale bars correspond to 500 nm.

Next, we tested whether RA27/3 immature particles accumulated intracellularly in HUVEC by using thin section transmission electron microscopy. RA27/3 infected Vero cells, which do accumulate infectious virions (Fig 6), served as a control. Viral replication complexes and viral particles were readily observed in Vero cells, but not detected in HUVEC (Fig 8). However, extracellular particles were detectable in the cell debris collected from the media of RA27/3 infected HUVEC cultures (Fig 8). Thus, no accumulation of immature vaccine viral particles was detected in HUVEC.

## Discussion

In this study we demonstrated that, similar to WT clinical RV isolates [18], RA27/3 vaccine strain can infect and persist in fetal endothelial cell cultures, but the establishment and characteristics of RA27/3 persistence were fundamentally different. Unlike WT strains, after high MOI infections the vaccine infected cultures initially went through a lytic crisis, which manifested as substantial CPE and death of approximately 50% of the cells in the monolayer. All cells were infected at the beginning and the end of the crisis, and therefore we concluded that the monolayers were mainly repopulated by infected cells after the crisis. What determines the fate of an individual cell after infection with vaccine virus (cell death or proliferation) is presently unclear. Since we used pooled HUVEC cultures from 20 donors, it is possible that cells from different individuals differ in sensitivity to RA27/3. However, we observed similar culture crisis in three different lots of HUVEC (data not shown), which makes this possibility unlikely.

We have shown previously that establishment of wtRV persistence in HUVEC did not involve the emergence of viral variants [18]. Comparison of the whole genome sequences of the stock and persisting RA27/3 vaccine virus also did not reveal selection of a particular virus variant after the crisis. Thus, heterogeneity in the vaccine virus stock, in which both cytopathic and non-cytopathic variants might be present, does not appear to be the cause of the crisis and establishment of persistence. However, single-cell sequencing of viral RNA *in situ* [30] might provide more detailed information on viruses in individual cells.

Our results demonstrated that, after the initial cycle of replication (Fig 1, 1 dpi), the yield of RA27/3 was comparable to that of RV-Dz (~5x10^5^ pfu/ml), then dropped by two logs drop after the crisis and remained at a constant low level for at least 30 days. Therefore, unlike HUVECs persistently infected with wtRV, which produced on average 1–5 infectious virions per cell every day [18], persistent RA27/3 HUVEC cultures produced on average only one infectious virion per 100 cells daily. Given that genomic RNA-to-PFU ratio was estimated to be ~40 for purified virions of both viruses (Table 2), the total yields were estimated to be 40–200 particles and 0.4 particles per cell daily in persistent RV-Dz and RA27/3 cultures, respectively. Since all cells were infected initially by RA27/3 and ~50% of them died, cell death can account for only 0.5-log reduction in RA27/3 yield. Other mechanisms must be involved in order to explain the additional 1.5-log reduction of vaccine virus titer after the crisis.

The ability of RA27/3 virus to establish persistent infection has been previously demonstrated in primary cultures of fetal joint tissues [31]. In agreement with our results, the authors detected viral antigens in a large proportion of cells and 2–3 logs lower virus yields compared to WT lab strains. However, they did not observe a spike of virus production soon after infection and a culture crisis. Unfortunately, the input multiplicity was not specified by the authors. Therefore, it is unclear if the lack of CPE in chondrocytes was because of the differential ability of RA27/3 to induce cell death in different cell types or because of the low MOI infections, similar to what we observed in HUVEC infected with an MOI of 0.05. Detailed investigations of fetal cell cultures of additional cell types might provide further insight into cytocidal properties of the RA27/3 vaccine.

For most steps of RV replication we were unable to identify differences sufficiently large to explain the 2-log difference in infectious virus production between persistent cultures of vaccine and WT viruses. Entry and egress of WT and vaccine viruses appeared to be equally effective, structural proteins were produced at the similar levels and no substantial differences were found in kinetics of viral RNA replication. Moreover, there was no accumulation of immature virions visible by TEM in infected cells and secreted virions were similar in protein composition and had compatible genomic RNA-to-pfu and genomic RNA-to-capsid ratios. By exclusion, these data suggest that virion assembly is the most likely step impaired in vaccine infected HUVEC. Unfortunately, the lack of understanding of molecular mechanisms of RV virion assembly and the rapid egress of mature virions from endothelial cells hampered a comparative analysis of vaccine and WT RV virion assembly in HUVEC. However, the observation that WT and vaccine viruses were produced at similar levels after the initial cycle of replication led us to think that the virion assembly block is not an intrinsic property of RA27/3, but was likely induced by a strain-specific cellular response to infection. Interferon-stimulated genes can block virus production at any stage of a virus replication cycle [32, 33]. Antiviral responses targeting assembly and morphogenesis of progeny virus have been documented for some viruses including HIV-1, influenza and respiratory syncytial viruses [34–37].

One of the novel results of this study is quantification of negative-strand (replication template) and positive-strand genomic RNA in individual infected cells by an *in situ* hybridization assay based on branch DNA technology. Surprisingly, we detected only a few negative-strand RNAs per cell (median values from 1 to 4) in both WT and vaccine infected HUVEC. This could explain why we were unable to detect replication complexes by TEM in RV-Dz [18] and RA27/3 infected HUVEC (Fig 7). For comparison, ~27,000 replication complexes each containing one or two molecules of negative-strand RNA were detected in BHK cells infected with Sinbis virus [38]. The average amounts of accumulated positive-strand genomic RNA were similar in the majority of cells infected with WT and vaccine viruses (~50 RNA copies/cell). Therefore, the proportion of negative-strand to positive-strand RNA was approximately 1:50, which is compatible to the previously determined value of 1:80 for the lab strain M33 in Vero cells [39].

Compared to wtRV, the major distinct characteristics of RA27/3 replication in HUVEC were the presence of elevated amounts of negative-strand RV RNA and RV dsRNA at the beginning of the crisis (Figs 4 and 5) and the accumulation of high amounts of positive-strand genomic RNA in a subset of infected cells during the crisis and persistence (Fig 3, Table 1). Why only a subset of HUVECs produced large amounts of RA27/3 RNA is not clear at this time. Since no dsRNA was detected in persistent cultures and the percentage of cell death (50–70%) during establishment of RA27/3 persistence were similar to the proportion of cells with detectable dsRNA amounts (58%) at the beginning of the crisis, it is reasonable to suggest that the accumulation of dsRNA triggered death of RA27/3 infected cells and culture crisis. Indeed, dsRNA was shown to be a strong inducer of apoptosis in HUVECs sensitized by interferon [40]. In addition, in surviving cells, RV dsRNA could be involved in inducing host responses that block virion assembly and lead to establishment of persistent cultures with low virus yield. Although in the past interferon response was postulated to be nonessential for establishment of RV persistence, convincing evidence is lacking [41, 42]. Alternatively, interferon response might be critical for establishment of persistent cultures of RA27/3 vaccine virus, but unnecessary for WT clinical strains. A consistent finding is that type 1 interferon expression is not absolutely required for the establishment of rubella virus persistence [43]. Consistent with these observations, we also find that type 1 interferon expression is not required for establishment of persistence of vaccine or WT RV in HUVECs (data not shown). We are currently examining additional details of the innate immune response to rubella virus infection in HUVECs.

In conclusion, the data presented here suggest that the mechanism(s) of establishment and maintenance of RV persistence in primary fetal endothelial cell cultures differ substantially for clinical strains and the RA27/2 vaccine virus. As we have shown earlier, wtRV does not induce any pathologic changes in HUVEC or interfere with cell metabolism resulting in survival of all infected cells, which divide and persistently produce high virus titers [18]. Here, we documented major differences between WT and RA27/3 in virus-host interactions, which might be depended on levels of dsRNA or interferon produced. We propose that the establishment of RA27/3 persistent cultures proceeds through several stages including 1) an initial cycle of replication comparable to wtRV followed by 2) the death of cells containing relatively high amounts of RV dsRNA during a crisis period and 3) continuing inhibition of virion assembly allowing a subset of cells to survive and give rise to a persistently infected culture.
